# Effects of spontaneous breathing anesthesia on postoperative cognitive dysfunction in elderly patients: a narrative review

**DOI:** 10.3389/fsurg.2026.1772706

**Published:** 2026-04-13

**Authors:** Tongxin Zheng, Lei Chen

**Affiliations:** 1 Thoracic Surgery Department, School of Clinical Medicine, Hebei University, Baoding, Hebei, China; 2 Department of Anesthesiology, the Affiliated Hospital of Hebei University, Baoding, Hebei, China

**Keywords:** clinical evidence, elderly patients, mechanism, POCD, review, spontaneous breathing anesthesia

## Abstract

Postoperative cognitive dysfunction (POCD) is a common and severe central nervous system complication following anesthesia in elderly patients, significantly increasing their medical burden and reducing quality of life. Conventional endotracheal intubation under general anesthesia may be a potential risk factor, while the associated effects of spontaneous-breathing anesthesia remain inconclusive. This paper elucidates the pathophysiological mechanisms of POCD, including neuroinflammatory cascades, cerebral oxygen metabolism imbalance, blood-brain barrier disruption, and the unique vulnerability of elderly brain tissue. It also analyzes the neuroprotective properties of spontaneous-breathing anesthesia, which optimizes cerebral oxygen supply-demand balance, reduces systemic and central inflammation, and modulates hypothalamic-pituitary-adrenal axis stress responses—demonstrating distinct mechanisms from conventional endotracheal intubation anesthesia. Existing clinical evidence indicates its cognitive protective effects in elderly patients during certain surgeries, though heterogeneity in outcomes across procedure types and methodological limitations in studies persist. Furthermore, this paper outlines key perioperative management points for this anesthetic technique, addresses related controversies, and identifies future research directions such as multimodal monitoring and individualized protocols, providing crucial guidance for perioperative brain health management in elderly patients.

## Key findings

- Elucidatesthe pathophysiological mechanisms of POCD
- Analyzes the neuroprotective properties of spontaneous-breathing anesthesia,
- Outlines key perioperative management points for this anesthetic technique
- Identifies future research directions

## What is known and what is new?

- KNOWN: the pathophysiological mechanisms of POCD; the neuroprotective properties of spontaneous-breathing anesthesia; key perioperative management points for this anesthetic technique
- NEW: future research directions

## What is the implication, and what should change now?

- Compared with tracheal intubation anesthesia, Spontaneous Breathing Anesthesia avoids the effects of airway mechanical stimulation and positive-pressure ventilation on cerebral blood flow, and mechanistically reduces the risk of POCD in elderly patients.

## 1 Introduction

Spontaneous Breathing Anesthesia (SBA) is defined as an anesthetic technique that takes maintaining or minimally interfering with the patient's spontaneous breathing as the core principle. It adopts supraglottic airway tools (e.g., laryngeal mask) to establish the airway, avoids endotracheal intubation and invasive mechanical ventilation control, and matches the appropriate sedation and analgesia depth according to the surgical needs to ensure the stability of the patient's respiratory and circulatory functions.

Postoperative Cognitive Dysfunction (POCD) is a serious central nervous system complication that occurs after anesthesia/surgery, and is characterized by cognitive decline, including symptoms such as memory loss, inattention, and difficulty in communication (1, 2). The condition is particularly common in elderly patients and may last for days, months, or even years, resulting in prolonged hospitalization, increased healthcare costs, increased mortality, and a significant reduction in quality of life (3, 4).

With the acceleration of population aging, the health problems of the elderly population are becoming more and more prominent. Numerous clinical studies and trials have confirmed that elderly patients may develop POCD after undergoing general anesthesia/surgery. This dysfunction significantly increases the healthcare burden of elderly patients and has a serious impact on their postoperative recovery and quality of life (5).

Traditional general anesthesia with tracheal intubation is a widely used anesthetic in surgical procedures. However, it has been suggested that this method of anesthesia itself or its related operations (e.g., tracheal intubation stimulation, mechanical ventilation parameter settings, etc.) may potentially affect the neurological system of patients, especially elderly patients, constituting a risk factor for postoperative cognitive decline. The exact mechanisms need to be explored in depth.

Although postoperative cognitive dysfunction is an important clinical problem in elderly patients, the mechanisms by which it occurs are still not fully elucidated (5). At the same time, there are significant research gaps regarding whether and how the SBA modality can mitigate the risk of postoperative cognitive dysfunction in elderly patients compared to conventional tracheal intubation anesthesia. It is of great clinical value to clarify the effects of SBAon postoperative cognitive function in elderly patients and its underlying mechanisms. To address these questions, This article summarizes the pathophysiological mechanisms of POCD, neuroprotective pathways of SBA, clinical evidence across surgical types, perioperative management strategies, controversies, and future research directions, aiming to provide a comprehensive and evidence-based reference for clinical practice.

## 2 Pathophysiologic mechanism of postoperative cognitive dysfunction

The occurrence of postoperative cognitive dysfunction (POCD) involves the interaction of multiple factors, and its core pathophysiological mechanisms include neuroinflammatory cascade response, imbalance of cerebral oxygen metabolism, disruption of the blood-brain barrier, and the special susceptibility of elderly brain tissue.

### 2.1 The central role of neuroinflammatory response

Neuroinflammation is a central driver of POCD. Surgical trauma and anesthesia activate the peripheral immune system, prompting the release of pro-inflammatory cytokines (e.g., IL-6) and triggering central neuroinflammation through the circulatory system (6). Microglia and astrocytes are activated to release inflammatory mediators such as tumor necrosis factor-α (TNF-α) and interleukin-1β (IL-1β), which directly damage neurons in the hippocampus—a brain region that is critical for learning memory (7, 8). Clinical evidence suggests that postoperative neuroinflammation is significantly associated with decreased cognitive test scores, and that the longer the duration of inflammation, the higher the risk of cognitive impairment (9). Neuroinflammation can also disrupt synaptic plasticity by downregulating N-methyl-D-aspartate receptor (NMDAR) expression, further impairing cognitive function (8).

### 2.2 Imbalance of cerebral oxygen supply and demand and microcirculation disorders

Cerebral oxygen metabolism disorder is the key link of POCD. Surgical stress can lead to impaired cerebral blood flow autoregulation, especially in elderly patients, which is prone to cerebral oxygen supply and demand imbalance (10). Microcirculatory disorders are manifested as inadequate cerebral capillary perfusion, which directly leads to neuronal energy failure (11). Mitochondrial dysfunction exacerbates oxidative stress and generates excess reactive oxygen species (ROS), causing neuronal DNA damage and apoptosis (10, 11). In addition, glucose transporter 1 (GLUT1) expression is down-regulated in cerebral microvessels, impeding glucose transport to the brain parenchyma, further limiting energy supply and accelerating cognitive decline (11, 12).

### 2.3 Disruption of blood-brain barrier integrity under stress response

Blood-brain barrier (BBB) disruption is a pathological hallmark of POCD. Surgical stress increases vascular permeability by activating the hypothalamic-pituitary-adrenal axis, leading to structural disintegration of the BBB (13). Abnormal expression of tight junction proteins (e.g., CLDN5) impairs the barrier function, and peripheral inflammatory factors (e.g., Aβ), immune cells, and toxic substances penetrate into the brain parenchyma (14, 15). Clinical studies have demonstrated that BBB damage markers such as S100β are significantly elevated in the cerebrospinal fluid of patients with postoperative delirium and are associated with prolonged hospitalization (12, 13). BBB damage also exacerbates the vicious cycle of neuroinflammation, forming a “peripheral-central” inflammatory transmission pathway (10, 14).

### 2.4 Special susceptibility of elderly brain tissues to POCD

The susceptibility of the aging brain to POCD stems from multiple pathologies: (1). **Vascular aging**: age-related cerebrovascular dysfunction diminishes cerebral blood flow reserve, and decreased microcirculatory clearance leads to accumulation of metabolic wastes (e.g., Aβ) (3, 12). (2). **Superimposed neurodegenerative pathologies**: Alzheimer's disease (AD)-related pathologies (Aβ plaques, tau protein tangles) and surgical stress have a synergistic effect on POCD. Plasma Aβ levels increase after surgery, and BBB dysfunction makes it easier for peripheral Aβ to enter the brain, accelerating neuronal damage (16, 17). (3). **Barrier function decline**: the expression of proteins such as CLDN5 and other proteins in the structure of the BBB decreases in the elderly, and the basal permeability increases, so that the barrier effect on inflammatory factors is significantly weakened (12, 15). (4). **Inadequate mitochondrial reserve**: the function of neuronal mitochondria declines with age, and the ability to cope with oxidative stress is reduced, exacerbating the surgery-induced energy crisis (11, 17).

In summary, POCD in the elderly is the result of the joint action of neuroinflammation, oxygen metabolism disorder, and BBB destruction in aging brain tissues, and its pathological mechanism presents significant age-dependent characteristics (3, 12, 17).

## 3 Neuroprotective properties of spontaneous breathing anesthesia

### 3.1 Dynamic effects on cerebral oxygen saturation

SBA may optimize the balance between cerebral oxygen supply and demand by maintaining physiological breathing patterns. It has been shown that mechanical ventilation (commonly associated with tracheal intubation anesthesia) is associated with inflammatory responses and apoptosis in the hippocampus (18). In contrast, techniques that preserve autonomic breathing significantly reduce serum levels of markers of neurologic injury and improve heart rate variability, suggesting better autonomic regulation and cerebral perfusion stability (18). In addition, reduced cerebrospinal fluid (CSF) production in older individuals may exacerbate impaired cerebral metabolic waste removal (19), and spontaneous respiratory patterns may indirectly support cerebral oxygen homeostasis by maintaining physiologic fluctuations in intrathoracic pressure and facilitating cerebral venous return and CSF kinetics.

### 3.2 Modulation of systemic/central inflammatory responses

SBAattenuates systemic and CNS inflammatory responses. Tracheal intubation anesthesia (e.g., sevoflurane) activates microglia and significantly elevates levels of pro-inflammatory factors IL-1β, IL-6, and TNF-alpha in the hippocampus (20, 21), and induces the expression of genes related to neuroinflammation (e.g., NOX2, NOX4) (22). Meta-regression analysis confirms a direct correlation between inhalation anesthesia in the immediate postoperative period and elevation of IL-1β, TNF-alpha (23). On the contrary, spontaneous breathing patterns may reduce peripheral inflammatory signaling to the center by avoiding airway instrument stimulation with mechanical tugging. Experiments have shown that inhibition of inflammatory pathways (e.g., IL-17A) improves anesthesia-related cognitive-behavioral deficits (22), indirectly supporting the importance of reducing inflammatory activation for neuroprotection.

### 3.3 Stress regulation of the hypothalamic-pituitary-adrenal axis

SBA may maintain HPA axis homeostasis by reducing surgical stress. Tracheal intubation anesthesia (e.g., sevoflurane, isoflurane) induces sustained hyperactivation of the HPA axis, which is manifested by activation of corticotropin-releasing hormone (CRH) neurons (24, 25) and dysregulation of cortisol secretion, which in turn exacerbates neuroinflammation and cognitive impairment (26). Excessive activation of the HPA axis and activation of microglial cells in the hippocampus form a vicious cycle that promotes neurodegeneration (27). Clinical studies have shown that HPA axis dysfunction is closely related to postoperative delirium and cognitive impairment (28). SBA by avoiding the stimulation of tracheal intubation, may reduce the stress response of the HPA axis and maintain the stability of cortisol rhythms, thus attenuating its toxic effects on neurons (28, 29).

### 3.4 Comparison of mechanistic differences with tracheal intubation anesthesia

The core mechanistic differences between SBA and tracheal intubation anesthesia are reflected in three aspects: (1). Inflammatory pathways: tracheal intubation anesthesia directly activates peripheral and central inflammatory cascade responses (e.g., IL-1β, TNF-α release) through physical stimuli and inhaled drugs (20, 23, 30), whereas the spontaneous breathing mode reduces such stimuli, which may reduce the risk of neuroinflammation. (2). HPA axis modulation: Tracheal intubation anesthesia significantly enhances CRH neuronal activity, leading to persistent HPA axis hyperactivity and cortisol dysregulation (25, 26), whereas autonomic respiration may maintain HPA axis feedback homeostasis by decreasing the intensity of surgical stress (31). (3). Cerebral oxygenation and metabolism: Mechanical ventilation may impair cerebral autoregulation, whereas autonomic respiratory techniques improve cerebral oxygen saturation by maintaining physiological respiratory variability, improving cerebral oxygenation monitoring indices and heart rate variability, and reducing hippocampal inflammation and apoptosis (32). Cohort studies further support that inhalation anesthesia significantly increases the risk of dementia in elderly patients compared to non-inhalation anesthesia (32), highlighting the impact of technique choice on neuroprognosis.

## 4 Comprehensive evaluation of clinical evidence

### 4.1 Evidence for cognitive function protection in orthopedic surgery

In the field of orthopedic surgery, randomized controlled trials of hip fracture surgery have shown no significant difference between general and regional anesthesia on the incidence of POCD. Assessment of cognitive outcomes at 24 hours, 3 days, and 7 days postoperatively showed that both anesthesia techniques had a similar impact on elderly patients, suggesting that anesthesia selection should be based on individualized factors rather than simply avoiding the risk of POCD (33). For elderly patients undergoing major orthopedic surgery, the Ketamine Intervention Study (POCK trial) explored the role of prophylactic medication on neurocognitive deficits, but the results are not yet clear (34). In addition, the Total Hip Arthroplasty (THA) study noted that opioid anesthesia may impede postoperative recovery, whereas opioid-exempt anesthesia (OFA) improves the quality of recovery in elderly patients (19).

### 4.2 Evidence for cognitive function protection in thoracic surgery

In thoracic surgery, non-intubated video-assisted thoracic surgery (NIVATS) is a typical clinical application of SBA,. A cohort study focusing on obese ILD patients (BMI≥30) confirmed that NIVATS under SBA achieved satisfactory perioperative outcomes, with no significant differences in postoperative complication rates, numerical pain rating scale (NPRS) scores and postoperative length of stay between obese patients and normal/overweight patients (*P* > 0.05), and the overall diagnostic yield reached 99% without conversion to tracheal intubation during the operation (35). This study indicated that obesity, which was previously regarded as a relative contraindication for non-intubated thoracic surgery, is not a dominant risk factor for SBA application in minimally invasive thoracic procedures. More importantly, SBA avoids tracheal intubation stimulation and positive pressure mechanical ventilation injury in thoracic surgery, which is consistent with its core neuroprotective mechanisms such as reducing systemic stress response and optimizing cerebral oxygen metabolism, and further provides real-world clinical evidence for the cognitive protection effect of SBA in thoracic surgery, especially for high-risk subgroups such as obese patients.

### 4.3 Analysis of heterogeneity of outcomes across surgery types

The type of surgery significantly affects the neuroprotective effect of SBA A meta-analysis of hip surgery showed that the difference between general versus regional anesthesia on the incidence of postoperative delirium (POD) or POCD was controversial, with conflicting findings from different studies (36). Studies of laparoscopic colorectal surgery have shown that maintenance anesthesia with remazolam reduces the risk of neurocognitive deficits in elderly patients, and that the effect is more pronounced at medium and high doses (37, 38). A network meta-analysis of elderly patients undergoing non-cardiac surgery found heterogeneity in the preventive effect of different anesthetic drugs on POCD (39). In addition, combined epidural-general anesthesia in major thoracic and abdominal surgery reduces the incidence of postoperative delirium (40).

### 4.4 Specific benefit characteristics of the elderly subgroup of patients

Elderly patients (≥60 years old), as a high-risk group for POCD, have specific benefit characteristics. A network meta-analysis of elderly patients undergoing non-cardiac surgery confirmed that anesthetic drug choice directly influences the incidence of POCD (39, 41). A randomized trial of orthopedic and pancreatic surgery showed better neurocognitive recovery in an elderly subgroup (≥65 years) receiving specific anesthetic regimens such as propofol or remazolam (42, 43). Intraoperative application of low-dose esketamine in elderly gastrointestinal oncology patients with preoperative anxiety reduces the risk of postoperative delirium and improves cognitive function (44). Probiotic intervention significantly reduces the incidence of POCD in elderly patients by suppressing the inflammatory response and modulating intestinal flora (4). Elderly (≥80 years) patients had an increased risk of mechanical ventilation withdrawal failure, but cognitive outcomes were not significantly different from the younger elderly group (60–79 years) (45).

### 4.5 Methodological limitations of existing randomized controlled trials

The current studies suffers from multiple methodological flaws. Data on 12-month cognitive decline in hip fracture surgery still relies on observational studies and there is a lack of randomized controlled evidence to support the long-term benefits of regional anesthesia (3, 46–48). Multiple randomized trials have not systematically monitored physiological markers (e.g., cerebral oxygen saturation, inflammatory markers), limiting in-depth resolution of mechanistic associations (49). This leads to Prolonged extubation time and increased incidence of postoperative chills in the high-dose group in anesthesia drug dose optimization studies (e.g., remazolam), but the effects on long-term cognitive function were not adequately assessed (38). Trials comparing anesthesia regimens (e.g., spinal vs. general anesthesia) in elderly patients with cognitive impairment have insufficient sample sizes and do not stratify analyses according to baseline cognitive status and further subgroup analysis (50). Most studies lacked standardized neurocognitive assessment tools and blinding and insufficient long-term follow-up data.

## 5 Technical maneuvers and perioperative management points

### 5.1 Drug selection and dose optimization strategies

The choice of medications for SBAneeds to take into account the minimization of respiratory depression and neuroprotection. Intravenous anesthetics such as remimazolam showed dose-dependent cognitive protection in elderly laparoscopic colorectal surgery, with the incidence of cognitive dysfunction on the third postoperative day in the low and medium-dose group (0.2–0.3 mg/kg/h) significantly lower than that in the high-dose group (0.4 mg/kg/h) (49). Ketamine (esketamine), an NMDA receptor antagonist, reduces the incidence of postoperative delirium with a single subanesthetic dose (0.5 mg/kg) in orthopedic surgery, but the long-term protection of cognitive function still needs to be verified (1, 49). A low dose (0.2 mg/kg) of esketamine in preoperatively anxious elderly patients improves 24-hour postoperative cognitive ratings score (42). A comparative study of the inhalation anesthetic sevoflurane versus propofol showed no significant difference in the effects on cognitive function and Alzheimer's disease-related biomarkers at 6 weeks postoperatively (51, 52). It should be noted that drug metabolism slows down in elderly patients, and it is recommended to titrate the dose individually based on body weight, hepatic and renal function to avoid postoperative cognitive risk caused by improper management of intraoperative anesthetic management (52–54).

### 5.2 Key indicators of respiratory function monitoring

Maintaining a balance between cerebral oxygen supply and demand is the core goal, and the following indicators need to be monitored dynamically:

1. **pulse oximetry (SpO₂)**: ≥95% should be maintained intraoperatively, especially in elderly patients, hypoxemia (SpO₂ < 90%) is significantly associated with postoperative cognitive impairment (55, 56).
2. partial pressure of end-expiratory carbon dioxide (EtCO₂): target range 35–45 mmHg).
3. Cerebral oxygen saturation (rSO_2_): real-time monitoring of local oxygen supply in the brain, timely detection of cerebral insufficiency, and guidance for adjustment of anesthesia regimen.

### 5.3 Prevention and management of common complications

1. Hypotension: Prevention: crystalloid supplementation 5–7 mL/kg prior to induction of anesthesia, α₂ agonists (e.g., dexmedetomidine) are recommended to maintain hemodynamic stability in elderly patients (57, 58).

Management: phenylephrine 50–100 μg intravenous push is preferred, and norepinephrine 0.05–0.1 μg/kg/min infusion is added for persistent hypotension (58, 59).

(1) Postoperative nausea and vomiting (PONV): dexamethasone 4–5mg + ondansetron 4mg preoperatively in high-risk patients (elderly women, non-smokers), avoiding inhalation anesthetics during surgery (60–62).
(2) regional anesthesia complications: Nerve injury prevention: ultrasound-guided puncture, limiting the amount of single-site local anesthetics (e.g., ropivacaine ≤ 3 mg/kg) (59, 60).

Management of anesthetic toxicity: in the presence of tinnitus or metallic taste, stop administration immediately and administer 20% fat emulsion 1.5 mL/kg intravenously (59, 63).

### 5.4 Safety and implementation framework

The clinical implementation of SBA requires the establishment of a standardized safety framework, including: ①Patient selection: priority is given to elderly patients with ASA grades I to III, no serious respiratory dysfunction, and good airway conditions, and patients with obstructive sleep apnea and severe pulmonary dysfunction are fully preoperatively evaluated; ② Remedial airway plan: prepare airway tools such as tracheal intubation and laryngeal mask during surgery, establish a rapid airway management process, and initiate airway remedial measures immediately when SpO 2 persists < 90%, EtCO 2 > 50 mmHg, or apnea exceeds 30 s; ③ Upgrade standard: clear indications for anesthesia conversion, if the patient has severe hemodynamic disturbances, intractable hypoxia/hypercapnia If the scope of surgery is expanded, it should be promptly converted to endotracheal intubation anesthesia.

## 6 Areas of controversy and unanswered questions

### 6.1 Controversy in defining the population of optimal indications

Currently there is significant controversy about the definition of the population for which SBA is indicated, especially for elderly patients with different underlying cognitive states and types of surgery. Several studies have shown unclear differences in the effects of spinal versus general anesthesia on postoperative delirium (POD) or cognitive dysfunction (POCD), especially in patients with preexisting preoperative cognitive impairments, including dementia (64). An analysis of elderly patients undergoing hip fracture surgery showed no statistically significant difference in the incidence of cognitive decline at 12 months postoperatively, regardless of whether general or regional anesthesia was used (29.7% vs. 25.4%) (46), suggesting that the choice of anesthesia modality may need to be more dependent on individual patient characteristics rather than a uniform standard. In addition, preoperative cognitive impairment has been shown to be an independent risk factor for postoperative delirium (65), but there is still a lack of consensus on how to accurately screen the population for the benefit of SBA accordingly. Controversy also focuses on elderly patients with comorbid neurodegenerative diseases (e.g., Alzheimer's Disease, whose perioperative cognitive risk requires special assessment, but existing guidelines have not yet clarified the strategies for optimizing anesthetic regimens in this population (65, 66).

### 6.2 Insufficient evidence of long-term cognitive protective effects

There is a lack of high-quality evidence supporting the protective effect of SBA on long-term cognitive function in elderly patients. Existing studies have mostly focused on short-term postoperative cognitive assessment (e.g., 7 days to 3 months), and there is a serious lack of data on follow-up at 12 months and beyond (59, 67). In a sub-study of a multicenter randomized trial, the incidence of “major cognitive decline” at 12 months in patients undergoing hip fracture surgery under general or regional anesthesia was 8.6% and 8.5%, respectively, which was not significantly different (OR: 1.0, 95% CI: 0.4–2.4) (46). In addition, a long-term cohort study of elderly patients undergoing non-cardiac surgery noted a higher incidence of dementia with general anesthesia (especially inhalation anesthesia) compared to regional anesthesia (3647.90 vs. 272.99/100,000 person-years) (66), but the results need to be further validated in randomized controlled trials (51). Notably, animal experiments suggest that anesthesia/surgery may accelerate brain aging through epigenetic modulation (56, 68), but studies of such mechanisms have not yet translated into clinical evidence of long-term cognitive protection in humans. There are no studies directly comparing the effects of SBA with conventional anesthesia on cognitive function over more than 1 year, resulting in inconclusive evidence of its long-term benefits (69, 70).

### 6.3 Synergistic effects with other brain protection measures

The synergistic effects of SBA with other brain protection strategies (e.g., pharmacological interventions, neuromodulation) are still an unresolved area. A small number of studies suggest that combined interventions may have potential: for example, a small dose of esketamine (0.25 mg/kg) given during the induction of anesthesia reduced the incidence of postoperative delirium and improved early cognitive function (62, 71), but this result was not evaluated in combination with SBA. The neuroprotective effect of ketamine may be achieved by inhibiting neuroinflammation (72), and its synergistic mechanism with SBA in modulating the hypothalamic-pituitary-adrenal axis stress response has not been explored (73). In the non-pharmacological field, repetitive transcranial magnetic stimulation (rTMS) and transcranial direct current stimulation (tDCS) have not been studied in conjunction with SBA, although they have shown potential for postoperative cognitive improvement (74, 75). In addition, perioperative probiotic interventions may reduce the risk of POCD by inhibiting inflammatory responses and modulating gut flora (76), while there is a gap in the effect of SBA on gut microecology. Existing synergistic studies face three major limitations: (1) most of them are single-measure effect analyses, and there is a lack of multimodal joint trials; (2) the mechanism studies are limited to animal models, and there is a lack of clinical translation; (3) there is no differentiation between different types of surgeries and patients' baseline risks (56, 77).

### 6.4 Intercentral heterogeneity in clinical implementation

In the typical clinical application scenarios of SBA such as unintubated thoracic surgery, current clinical practice shows significant inter-center heterogeneity in patient selection and perioperative implementation. There are large differences in patient inclusion standards (78, 79), airway management strategies (80, 81), anesthetic drug combinations (81, 82), and perioperative monitoring indicators (81, 83) among different medical centers, which makes it difficult to compare the results horizontally and limits the accurate assessment of the neuroprotective effects of SBA. This current situation directly confirms that standardized operating protocols are the premise of conducting multicenter neurocognitive endpoint comparison studies, and are also a key issue to be addressed in future clinical research and practice. A national survey of 55 Italian thoracic surgery centers further verified the complexity of this heterogeneity (84). The results showed that although 78% of the centers carried out SBA-based non-intubated thoracic surgery (NITS), only 38% of them extended the technique to parenchymal lung surgery such as lung biopsy, with an average of only 8.25 cases per year. There were obvious differences in the judgment of indications and contraindications among centers: 70% of anesthesiologists regarded obesity (BMI ≥ 30) as a contraindication to SBA, while only 52% of surgeons held the same view, and there was no consensus on whether preoperative non-invasive ventilation or expected difficult airway should be excluded. In terms of anesthetic management, the regional anesthesia techniques adopted by various centers were diverse, including paravertebral block (37%), serratus anterior plane block (32%), epidural block (32%), etc., and the sedation schemes were mainly propofol (84%) and remifentanil (69%), but there was no unified standard for drug dosage and monitoring indicators. In addition, 31% of the centers reported the experience of converting SBA to general anesthesia, but the conversion triggers and operation processes varied greatly among centers.

This heterogeneity not only exists in the selection of patients and technical operation, but also in the cognitive level of clinical staff: centers with rich SBA experience tend to recognize fewer contraindications and believe that the benefits of neuroprotection and reduced stress response outweigh the potential risks, while centers without relevant experience are more worried about airway management and intraoperative complications. These differences directly lead to the difficulty in accumulating high-quality consistent evidence on the neuroprotective effect of SBA, and further highlight the urgency of formulating evidence-based standardized operation procedures and carrying out multicenter collaborative research.

## 7 Future research directions

### 7.1 Application of multimodal neuromonitoring technology

Current research needs to further integrate real-time neurological function monitoring techniques to dynamically assess the effects of SBAon cerebral oxygen metabolism and neurophysiological activity. Cerebral oxygen saturation (rSO₂) monitoring combined with quantitative electroencephalography (qEEG) can capture the intraoperative state of cerebral oxygen supply/demand balance and changes in neural oscillatory patterns, which can provide a basis for optimizing the depth of anesthesia (57). The combination of near-infrared spectroscopy (NIRS) and transcranial Doppler ultrasound (TCD) can be used to synchronize the assessment of cerebral hemodynamics and microcirculatory function, and to reveal the mechanism of cerebral perfusion regulation by SBA in elderly patients (85). In the future, a multimodal monitoring platform needs to be developed to integrate hemodynamic, metabolic, and electrophysiological parameters to establish an early warning model to reduce the risk of postoperative neurocognitive impairment (86).

### 7.2 Biomarker-guided individualized anesthesia protocols

Inflammatory factors (e.g., IL-1β, IL-6) and markers of neurological injury (e.g., tau protein, neurofilament light chain) were significantly elevated in patients with postoperative cognitive dysfunction (POCD), suggesting that they could be used as biomarkers to predict neuroprotective effects of SBA (87). Metabolomics analysis revealed that reduced levels of S-methyl-5-adenylate (MTA) were associated with the risk of delayed postoperative neurocognitive recovery (dNCR), providing a new target for individualized intervention (87, 88). Gut flora-brain axis studies have shown that probiotic interventions reduce neuroinflammation and improve cognitive function by modulating flora metabolites (68). In the future, multi-group biomarker profiles (genomic, proteomic, metabolomic) need to be established, combined with machine learning algorithms to predict differences in patient response to SBA, to guide anesthesia drug selection and dose optimization (89).

### 7.3 Development and validation of standardized SBA operating procedures (SOP)

Formulate SOP for SBA in different surgical types (thoracic, laparoscopic, orthopedic) based on existing evidence, including patient selection criteria, drug combination schemes, monitoring indicators, and emergency conversion processes. Conduct multicenter validation studies to reduce inter-center heterogeneity and improve the reproducibility of clinical results.

### 7.4 Bridging pathways between mechanistic research and clinical translation

Basic research needs to deeply explore the molecular mechanisms by which SBA regulates neuroinflammation and blood-brain barrier integrity. Animal experiments have confirmed that anesthesia/surgery induces neuronal hyperexcitability and synaptic loss through activation of the endoplasmic reticulum unfolded protein response (UPR), and that targeted intervention of the UPR pathway improves cognitive function (90). Epigenetic studies have revealed the involvement of histone modifications and non-coding RNA regulation in the development of POCD, providing a basis for the development of epigenetic therapeutics (89, 91). Clinical translation requires the establishment of standardized SBA operating procedures and validation of mechanistic findings through multicenter randomized controlled trials (RCTs). Focus on optimizing anesthesia protocols for elderly subgroups (e.g., those with comorbid mild cognitive impairment) and conducting long-term follow-up to assess the sustained protective effect of SBA on cognitive function (66, 92).

## 8 Summary and core conclusions

### 8.1 Summary of mechanistic advantages of SBA

SBA exerts neuroprotective effects through multiple pathways. It maintains a physiological breathing pattern and significantly optimizes the balance of cerebral oxygen supply and demand. The technique effectively inhibits systemic and central neuroinflammatory responses, reduces the release of pro-inflammatory factors (e.g., IL-1β, IL-6), and attenuates microglial cell activation, thus alleviating the damage of neuroinflammation on cognitive function (72, 93). At the same time, SBA significantly reduces the intensity of surgical stress response, protects the integrity of the blood-brain barrier, and reduces the entry of neurotoxic substances into the brain by regulating the hypothalamic-pituitary-adrenal axis (67). Compared with tracheal intubation anesthesia, it avoids the effects of airway mechanical stimulation and positive-pressure ventilation on cerebral blood flow, and mechanistically reduces the risk of postoperative cognitive dysfunction (POCD) in elderly patients (75, 94).

### 8.2 Recommendations for current clinical practice

Based on the available evidence, SBA may be considered in selected elderly patients after careful preoperative assessment Drug selection needs to be individualized: maintenance anesthesia with a moderate dose of remazolam (e.g., 0.2–0.3 mg/kg/h) can balance cognitive protection and safety, avoiding the risk of delayed awakening and chills caused by high doses (95, 96); induction with a low dose of esketamine (0.25 mg/kg) can reduce the incidence of postoperative delirium (77, 97). Intraoperative monitoring of cerebral oxygen saturation (rSO_2_), end-expiratory carbon dioxide (EtCO_2_), and respiratory rate is required continuously to maintain PaCO_2_ in the physiologic range (35–45 mmHg) to safeguard cerebral perfusion. For those with comorbid sleep apnea or reduced lung function, an emergency airway management plan should be available to guard against hypercapnia and hypoxic events (66).

There are certain limitations to the certainty of evidence in this study: most of the existing clinical studies are short- and medium-term follow-up, and high-quality evidence of long-term cognitive benefits is insufficient; the results of different types of surgery are heterogeneous, and some studies have small sample sizes and methodological defects; The synergy effect of SBA and other brain protection measures has not been fully verified. In clinical application, it is necessary to fully consider the individual characteristics of patients such as age, underlying disease, and type of surgery to weigh the benefits and risks.

### 8.3 Implications for perioperative brain health management in elderly patients

The use of SBA highlights the multidimensional nature of perioperative brain health management. Preoperative baseline cognitive screening (e.g., MoCA scale) needs to be established to identify high-risk individuals (98, 99). Intraoperative integration of neurofunctional monitoring (e.g., EEG alpha power, cerebral oxygen saturation) allows real-time assessment of brain protection (100, 101). Neuroprotective strategies should be continued in the postoperative period: probiotic interventions reduce neuroinflammation and decrease the incidence of POCD by modulating the intestinal flora (49, 75); Repetitive transcranial magnetic stimulation (rTMS) in the early postoperative period may improve cognitive recovery (77, 102). Future focus needs to be on exploring biomarker (e.g., serum MTA, SIRT1) guided individualized anesthesia protocols and the construction of multimodal brain health management pathways (103–105).
